# Center of Biomedical Research Excellence in Matrix Biology: Building Research Infrastructure, Supporting Young Researchers, and Fostering Collaboration

**DOI:** 10.3390/ijms21062141

**Published:** 2020-03-20

**Authors:** Julia Thom Oxford, Ken A. Cornell, Jared J. Romero, Diane B. Smith, Tracy L. Yarnell, Rhiannon M. Wood, Cheryl L. Jorcyk, Trevor J. Lujan, Allan R. Albig, Kristen A. Mitchell, Owen M. McDougal, Daniel Fologea, David Estrada, Juliette K. Tinker, Rajesh Nagarajan, Don L. Warner, Troy T. Rohn, Jim Browning, Richard S. Beard, Lisa R. Warner, Brad E. Morrison, Clare K. Fitzpatrick, Gunes Uzer, Laura Bond, Stephanie M. Frahs, Cynthia Keller-Peck, Xinzhu Pu, Luke G. Woodbury, Matthew W. Turner

**Affiliations:** 1 Center of Biomedical Research Excellence in Matrix Biology, Boise State University, Boise, ID 83725, USA; 2 Department of Biological Sciences, Boise State University, Boise, ID 83725, USA; 3 Biomolecular Research Center, Boise State University, Boise, ID 83725, USA; 4 Department of Chemistry and Biochemistry, Boise State University, Boise, ID 83725, USA; 5 Division of Research, Boise State University, Boise, ID 83725, USA; 6 Department of Mechanical and Biomedical Engineering, Boise State University, Boise, ID 83725, USA; 7 Department of Physics, Boise State University, Boise, ID 83725, USA; 8 Micron School of Materials Science and Engineering, Boise State University, Boise, ID 83725, USA; 9 Department of Electrical and Computer Engineering, Boise State University, Boise, ID 83725, USA

**Keywords:** extracellular matrix, matrix biology, tissue regeneration, development, external advisory committee, mentored career development, research infrastructure, shared core facilities, pilot project program

## Abstract

The Center of Biomedical Research Excellence in Matrix Biology strives to improve our understanding of extracellular matrix at molecular, cellular, tissue, and organismal levels to generate new knowledge about pathophysiology, normal development, and regenerative medicine. The primary goals of the Center are to i) support junior investigators, ii) enhance the productivity of established scientists, iii) facilitate collaboration between both junior and established researchers, and iv) build biomedical research infrastructure that will support research relevant to cell–matrix interactions in disease progression, tissue repair and regeneration, and v) provide access to instrumentation and technical support. A Pilot Project program provides funding to investigators who propose applying their expertise to matrix biology questions. Support from the National Institute of General Medical Sciences at the National Institutes of Health that established the Center of Biomedical Research Excellence in Matrix Biology has significantly enhanced the infrastructure and the capabilities of researchers at Boise State University, leading to new approaches that address disease diagnosis, prevention, and treatment. New multidisciplinary collaborations have been formed with investigators who may not have previously considered how their biomedical research programs addressed fundamental and applied questions involving the extracellular matrix. Collaborations with the broader matrix biology community are encouraged.

## 1. Introduction

The purpose of this article is to inform the extracellular matrix (ECM) biology research community about the establishment of a new center for matrix biology research that can serve as a resource for investigators interested in disease-related and developmental biology aspects of matrix biology research. The National Institutes of Health (NIH) Centers of Biomedical Research Excellence (COBRE) program strengthens biomedical or behavioral research capacity in institutions from Institutional Development Award (IDeA)-eligible states. The COBRE program includes three phases. COBRE Phase I provides support to develop research infrastructure and to foster independence of junior investigators. COBRE Phase II continues the progress toward building an independent research center that is competitive for support from NIH or other funding agencies. The COBRE Phase III awards (Transitional Centers) provide support for maintaining COBRE research cores developed during phases I and II that are essential for ongoing basic, clinical, translational, and/or community-based research at the institution. In addition, a key role for Phase III awards is to sustain a collaborative, multidisciplinary research environment for research pilot projects as well as mentoring and training opportunities.

Here, we report on the COBRE in Matrix Biology. During Phase I, the program provided the opportunity to build infrastructure for biomedical research and to link researchers from multiple disciplines to carry out convergent science in research areas that are important to the ECM research community. Improvements to research infrastructure strengthened the biomedical research capacity established by the cohesive statewide NIH IDeA Network for Biomedical Research Excellence (INBRE) program, the Idaho State Board of Education, and the emerging research strength in matrix biology at Boise State University. Specific programmatic goals are presented in Table 1.

The programmatic goals outlined in Table 1 provided support and opportunities for biomedical researchers through shared research infrastructure, access to instrumentation, and mentoring. The outcome of the NIH COBRE in Matrix Biology Phase I support has been a sustainable change in the biomedical research culture at Boise State University, an institution of emerging excellence, thereby empowering researchers to address national health challenges. Investigators interested in becoming involved in the center are encouraged to contact the director and the corresponding author or use the website https://www.boisestate.edu/brc-cobre/.

## 2. Matrix Biology and the Extracellular Matrix

The thematic focus of Matrix Biology builds upon an emerging strength at Boise State University. Cells of the body exist in three-dimensional scaffolding called the ECM, which surrounds the cell and contains nutrients, proteins, and other molecules necessary for living. The matrix holds together the millions of cells that make up blood vessels, organs, skin, and all tissues of the body. The center focus includes molecular and cell biology of cell attachment and the ECM, cell surface proteins mediating cell-matrix interactions, and composition, structure, assembly, remodeling, and function of the matrix. The ECM is a key structural and functional regulator of organ and tissue function. Researchers in this arena examine how the ECM gives tissues their unique properties and determine how the local pericellular environment controls cell signaling and cellular responses to the local environment. It is possible, for example, to engineer extracellular matrices to improve post-injury wound healing by promoting tissue regeneration (as with stem cell differentiation or pattern formation) or by preventing scar formation. Central to matrix biology study is the critical role the ECM plays in both healthy and diseased tissue. Matrix biology naturally lends itself to interdisciplinary collaborations and a convergent problem-solving approach. For example, the theme has relevance in biomechanical and tissue engineering, liver physiology, cardiovascular biology, cancer progression, infectious diseases and host–pathogen interactions, neurobiology, developmental biology, and cell signaling.

The shared core facilities include imaging, proteomics, bioinformatics, tissue, and animal models to support the career development of junior investigators and the growth of biomedical research. The common thread uniting such research projects is the essential nature of the matrix in understanding the pathophysiology and providing new knowledge that will lead to improved diagnostics, preventions of disease progression, and therapeutic strategies for repair and regeneration of tissues. Investigators interested in access to the shared core facilities are encouraged to contact the Biomolecular Research Core here https://www.boisestate.edu/brc-cobre/biomolecular-research-core-2/.

Because matrix biology impacts nearly every physiological system and plays a critical role in numerous disease states, the COBRE for Matrix Biology fosters future research growth well beyond the initial research projects. The Center supports participation from both faculty and students who show academic excellence and talent in biomedical research and who are members of groups typically underrepresented in biomedical fields. To expand the depth and the breadth of the impact of biomedical research, it is imperative that skills and viewpoints of a diverse population are included in the research agenda.

## 3. Junior Investigator Career Development

A top priority for the Center is to provide financial support to junior faculty for individual projects that lead to subsequent NIH R01 or R01-like research funding. Junior investigators are usually assistant professors that develop independent research programs and compete successfully for independent R01-level funding by working with a mentor. The junior investigators are appointed for 2–3 years, during which they are expected to meet professional milestones. Upon graduation from the program, former junior investigators remain associated with the Center as research collaborators and future research mentors, transferring their experience to new junior investigators. New junior investigators are recruited through national and international searches, and the programmatic support can be a useful tool for building the ranks of new biomedical researchers in academic departments. Access to shared core facilities is provided for all junior investigators affiliated with the Center. Internally awarded pilot projects support smaller research projects proposed by other Center-associated faculty members.

Junior investigators maintain individual research projects and are responsible for meeting specific research aims. They must also meet annual milestones such as working with their mentor, publishing two manuscripts per year, attending and presenting at one national or international conference each year, and submitting an R01-like grant application before the end of the second year of funding. These prescribed annual milestones are designed to increase competitiveness for external funding. Each junior investigator has a mentor and works in full compliance with all applicable federal policies, rules, and guidelines for research involving human subjects, vertebrate animals, and biohazards. Mentors are faculty members that are established investigators who have maintained independent research laboratories and have proven track records in biomedical research productivity and extramural support. Grant writing workshops are provided annually as a component of the career mentoring program.

The COBRE evaluator from the administrative core collects data on the activities of the junior investigators as they work toward meeting specific metrics of career development. Metrics for each investigator include:

- Number of publications in peer-reviewed journals
- Presentations at national and international venues
- Patents and copyrights as a result of the COBRE in Matrix Biology
- Project investigator honors
- Grant submissions during the reporting period
- Awarded research support in addition to COBRE funding
- Highlights of the project investigator’s research group during the previous year
- Participation as a reviewer in the peer-review process
- Use of shared core facilities for research projects
- Graduate and undergraduate students involved in the project
- Unanticipated outcomes, problems, or pitfalls for which assistance can be offered
- Effectiveness of the mentor–mentee relationship
- Effectiveness in supporting inter-college partnerships
- Participation in growing the college and regional matrix biology network

## 4. Student Training

Doctoral programs offer graduate students education and research training opportunities in matrix biology. The Biomolecular Sciences Program, the Materials Science and Engineering Program, and the Biomedical Engineering Program work with the Matrix Biology COBRE to coordinate resources for the benefit of doctoral students. To recruit and retain highly talented graduate students, the COBRE provides nationally competitive research assistantships for the most promising applicants. Recognizing the essential role that graduate students will play in the next generation of biomedical research and acknowledging the need for diverse teams to tackle difficult questions, students from disadvantaged backgrounds and underrepresented groups are encouraged to apply.

## 5. Evaluation and Assessment

Assessment is essential for the evaluation of the COBRE program as a whole, individual researchers, research infrastructure, faculty development, and how well the COBRE enables researchers to attain independent grant support. Results of assessment and evaluation enable systematic and ongoing improvement. The center coordinate assessment, gather programmatic information, compile the annual report, then distribute it to the external advisory committee for evaluation.

Programmatic evaluation is carried out to assess overall center organization and attainment of goals and benchmarks. Formative and summative evaluations are prepared using an online reporting system. Benchmarks for COBRE impact on recruitment and retention of outstanding faculty and students are carried out by collecting information during support and after leaving the program. Other measures of progress include evidence of scholarly activities including publications and presentations, providing research experiences to students, impact on the community, attending and presenting research findings at scientific meetings and conferences, submission of external grant applications to NIH and other federal and non-federal agencies, and award acquisition.

## 6. External Advisory Committee

The Center of Biomedical Research Excellence in Matrix Biology is guided by an external advisory committee (EAC). The role of the EAC is to advise the director, assess the sustainability of the Center annually, and participate in appointments of new junior investigators. The composition of the EAC is designed to ensure the fulfillment of the goals and the broad scientific impact of the Center. The EAC comprises scientists with national and international reputation in their field. The membership of the committee includes distinguished scientists and individuals with specific expertise directing complex center programs. They offer expertise in matrix biology research as well as junior investigator mentorship and the development of institutional and research center infrastructure. The committee includes four members that provide constructive feedback to the director for scientific, administrative, and other matters that arise. Meeting twice a year to review scientific progress of the COBRE in Matrix Biology, members also encourage and assist faculty development and mentoring, identify resources, and evaluate center development, individual research project progress, and the overall COBRE program.

## 7. Building a Multidisciplinary Network of Matrix Biology Investigators

**Figure 1:**
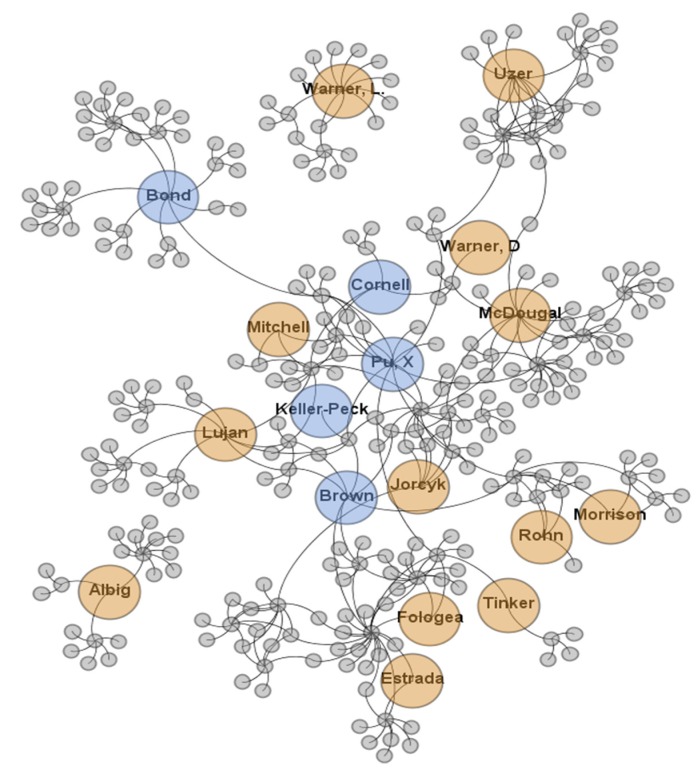
Co-authorship within the Center. This visual interpretation of the Center highlights 13 junior investigators (orange) and Biomolecular Research Core research scientists (blue) among 303 total authors affiliated with the Centers of Biomedical Research Excellence (COBRE) in Matrix Biology by citing the grant in their publications. Each node represents an individual author and edges (connections) represent co-authorship. Nodes repel each other like charged particles, while edges attract their nodes, like springs, creating a movement that converges to a balanced state. The final configuration provides a visual representation that shows that Core scientists localize centrally with a high indegree (edges between nodes) and connectedness. Nodes with high outdegree are located more peripherally. Source of bibliographic data: National Institutes of Health (NIH) reporter retrieved June 2018. The network was generated using Gephi visualization software [1,2,3,4].

A robust multidisciplinary network of matrix biology investigators are involved in the COBRE, including junior investigators and scientists at various senior stages of their careers. Those more senior in their careers provide scientific leadership for the Center and serve as mentors for junior faculty. A pilot project program is available to stimulate matrix biology research by new investigators and may serve as an onramp to the junior investigator program. It also serves to support senior investigators launching new lines of inquiry in the field of matrix biology. When a junior investigator graduates from the program through acquisition of independent R01 or R01-like funding, new investigators are appointed and move into the mentored research career development position. Mentors, mentees, and research scientists are encouraged to collaborate and publish together, which further expands the collaborative research network to include more senior investigators. A visual interpretation of the network of matrix biology is demonstrated through co-authorship analysis, as shown in Figure 1.

To foster added interdisciplinary interactions between investigators, the Center hosts weekly Research Grand Rounds, during which research presentations, informal discussions, and networking takes place. Research Grand Rounds include work-in-progress presentations to other matrix biology researchers, summaries of technical advances, and seminars on equipment capabilities organized by core personnel. Annually, the Center hosts a symposium, inviting speakers to present their work to coworkers and collaborators. The network interactions are furthered by additional workshops, and Lunch-and-Learn training opportunities are provided by core scientific staff that are open to faculty, postdoctoral fellows, students, and other investigators at nearby colleges and universities.

## 8. Shared Research Core Facilities 

The Center includes two cores: the Biomolecular Research Core and the Biomedical Research Vivarium Core. The two cores provide instrumentation and expertise that complements existing facilities. The Biomolecular Research Core supports instrumentation by service contracts and moderate user fees to sustain the core. The cores are supervised by experienced scientists, and daily operations (user scheduling, user training, and equipment maintenance) are carried out following clearly defined and web-accessible standard operating procedures.

The Center facilitates the study of biomolecules, with an emphasis on proteins and protein interactions. It maintains comprehensive instrumentation and facilities ideal for characterizing biomolecules and their role in a variety of biochemical processes. To perform their research, all investigators must be able to generate and analyze biological information. Therefore, the Biomolecular Research Core provides research support in histology, imaging and microscopy, genomics, proteomics, bioinformatics, biomechanics, cell and tissue culture, modeling, simulation, and data analysis. Technical staff maintains, operates, and provides training for all instrumentation in the core facility as well as the technical expertise for data management and data analysis required for competitive, multidisciplinary biomedical research. Matrix Biology investigators are encouraged to contact the Biomolecular Research Core by accessing https://www.boisestate.edu/brc/.

Instrumentation training workshops provide information on approaches and methods for data management, the development of methods for multidisciplinary research and resource sharing, and a framework for secure and confidential data sharing. Robust network connectivity facilitates research collaboration and resource sharing as well as access to education and training programs for graduate programs in Biomolecular Sciences, Materials Science and Engineering, and Biomedical Engineering that further national efforts to strengthen the biomedical research workforce.

The Biomedical Research Vivarium (BRV) provides care and housing for research animals essential to the research of the Center. The facility is available to researchers as a central animal research facility for work with mice and rats as well as zebrafish. Investigators are invited to attend seminars on key topics related to model systems used in biomedical research. Surgical assistance, breeding services, disease screening, and procedural training are provided by the BRV. The facility provides rooms for procedures, surgeries, cage and rack washing, necropsy, and storage. Per diem rates have been established and are reviewed each year. Standard operating procedures (SOP) are established, and a Vivarium Handbook is available for internal and external customers. The vivarium is administered by the Division of Research, overseen by the Office of Research Compliance. The institution has an established Institutional Animal Care and Use Committee (IACUC) that reviews all research, teaching, and testing and ensures compliance with U.S. Public Health Service (PHS) Policy and the Animal Welfare Act. The animal research program is registered with the United States Department of Agriculture and is assured with PHS.

## 9. Discussion and Future Directions

The COBRE Program offers the opportunity to build infrastructure for biomedical research and to link researchers from multiple disciplines to carry out convergent science in research areas of matrix biology. This reflects national discussion and investment that has identified a critical need for more extensive matrix biology research. For example, the Health and Human Services executive department recently released the report, “2020: A New Vision—A Future for Regenerative Medicine” [5], which called particular attention to tissue engineering and regenerative medicine.

The Center of Biomedical Research Excellence in Matrix Biology emphasizes a field of research with the potential to impact diverse national and international health needs. Because of the fundamental nature of research into the interaction of cells and their ECM, advances resulting from work carried out in the Center have the potential to translate improvements in many applications involved in patient care. Development of treatments for liver fibrosis, new developments in tissue regeneration, improved strategies for repair of injuries to tendons and ligaments, regulation of new blood vessel formation, maintenance of the circulatory system for the prevention of cardiovascular disease, and matrix-based approaches for the prevention of cancer progression are but a few of the anticipated outcomes resulting from the COBRE in Matrix Biology.

Junior investigators in the COBRE in Matrix Biology will ultimately contribute to the understanding and the treatment of numerous medical conditions with an enormous societal and economic impact to human health. Matrix biology is gaining prominence for its key role in organ structure, regulation, and function. In addition, researchers have currently identified at least 31 human diseases that occur as a result of direct genetic defects in ECM proteins. An emerging crossroads between matrix biology research and medical practice has created a unique opportunity to establish bench-to-bedside-to-bench translational matrix research as a pathway for improving quality of life.

Matrix biology research translates into several major focus areas: vascular biology and angiogenesis; inflammation and tumor microenvironment; cancer progression and metastasis; genetic and acquired diseases; organ fibrosis; tissue engineering; and stem cell biology. Key to the study of matrix biology is the understanding of critical roles of the ECM during tissue patterning and development (signaling during morphogenesis) and disruption of tissue structure and function during pathogenesis.

Future research directions and the efforts of investigators will contribute to establishing a multi-disciplinary research center by spanning traditional boundaries between disciplines. The center will foster cohesive interactions among scientists, promoting excellence and facilitating collaborations toward a convergence point. Capitalizing on emerging strengths in matrix biology, nanostructured biomaterials, tissue engineering, regenerative medicine, tissue repair, and replacement technologies will promote excellence in research and research training. Translation of these approaches will result in novel therapies, cell-based treatments, tissue replacement products, and tissue regeneration and repair. Development of scientific expertise in the biology of the cellular microenvironment, adult stem cells and their niches, nanostructured matrix scaffolds, 3-D tissue constructs, and bioreactors will promote this research effort in matrix biology.

The emphasis on translating regenerative medical technologies to practice and industry will attract researchers, industry, and investors to develop novel therapies and tissue replacement products. The Center is in an excellent position to develop a robust program in matrix biology, tissue engineering, and regenerative medicine as well as achieve national and international prominence in this area.

The COBRE in Matrix Biology allows for the competitiveness of researchers through access to core facilities, trained staff, seminars, training, and workshops. The Center serves as a focal point for interdisciplinary, multidisciplinary, multi-scale experimental, and computational investigations to develop useful products based on matrix biology such as nanostructured scaffolds and tissue replacement products.

Investigators interested in pursuing collaborative matrix biology research are encouraged to contact the Center of Biomedical Research Excellence in Matrix Biology at Boise State University. Additionally, the center operates a fee-for-service core facility that is available to the broader matrix biology research community. Further, interest in Matrix Biology research can be fostered by involvement in the American Society for Matrix Biology (www.ASMB.net), with which Center investigators participate. The International Society for Matrix Biology (https://ismb.org) supports similar work. Many national societies exist to support collaboration among investigators in the field of Matrix Biology such as the British Society for Matrix Biology, the Danish Society for Matrix Biology, the Dutch Society for Matrix Biology, the Finnish Connective Tissue Society, the French Society for Matrix Biology, the German Society for Matrix Biology, the Hellenic Research Club for Connective Tissue and Matrix Biology, the Italian Connective Tissue Society, Matrix Biology Ireland, the Swiss Society for Matrix Biology, the Canadian Connective Tissue Society, the Chinese Society of Matrix Biology, the Japanese Society for Matrix Biology and Medicine, and the Matrix Biology Society of Australia and New Zealand (https://ismb.org/national-societies/).

## Figures and Tables

**Table 1:** Specific goals of the Center of Biomedical Research Excellence in Matrix Biology.

Goal	Programmatic Description
Establish a critical mass of multidisciplinary investigators in Matrix Biology	Matrix Biology thematic focus Mentored research support for junior investigators Recruitment of new investigators Multidisciplinary networking Pilot project program
Consolidate and expand biomedical research core capabilities	Biomedical Research Vivarium Core Biomolecular Research Core (shared instrumentation)
Develop and maintain a significant and productive research program	Administrative Core for oversight and support Mentored junior investigator program Access to research instrumentation and facilities External Advisory Committee Steering Committee Recruitment of new investigators Pilot project program Annual program evaluation
Collaborate with existing programs	Leverage current infrastructure to reduce costly duplication Collaborate with Idaho IDeA Network for Biomedical Research Excellence (INBRE) program Maintain access to existing network of researchers
Expand student research training opportunities	Biomolecular Sciences doctoral program Materials Science and Engineering doctoral program Biomedical engineering doctoral program Training in matrix biology research techniques

## Abbreviations

COBRE	Center of Biomedical Research Excellence
IDeA	Institutional Development Award
INBRE	IDeA Network for Biomedical Research Excellence
NIH	National Institutes of Health
ECM	Extracellular Matrix
EAC	External Advisory Committee
IACUC	Institutional Animal Care and Use Committee
SOP	Standard Operating Procedure
PHS	Public Health Services
USDA	United States Department of Agriculture
